# Co-occurrence of Convergence Insufficiency and Cognitive Impairment in Parkinsonian Disorders: A Pilot Study

**DOI:** 10.3389/fneur.2019.00864

**Published:** 2019-08-09

**Authors:** Samantha K. Holden, Erin Van Dok, Victoria S. Pelak

**Affiliations:** ^1^Department of Neurology, University of Colorado School of Medicine, Aurora, CO, United States; ^2^Department of Ophthalmology, University of Colorado School of Medicine, Aurora, CO, United States

**Keywords:** convergence insufficiency, ocular motility, diplopia, Parkinson's disease/Parkinsonism, progressive supranuclear palsy, cognitive disorders

## Abstract

**Background:** Convergence insufficiency (CI) in parkinsonian conditions causes disabling visual symptoms during near tasks and usually manifests as double vision. Since double vision is more common in patients who report cognitive symptoms, we sought to determine if symptomatic CI, as opposed to asymptomatic CI, could serve as a marker of cognitive impairment in parkinsonian disorders.

**Methods:** Twenty-four participants with parkinsonian disorders (18 Parkinson's disease, 5 progressive supranuclear palsy, 1 multiple system atrophy) and objective findings of convergence insufficiency on neuro-ophthalmologic examination were included. Subjective visual symptoms and cognitive complaints were recorded, and the Self-Administered Gerocognitive Examination was used as a global cognitive screening measure.

**Results:** 54.1% of parkinsonian participants had cognitive impairment, but there were no significant differences in the degree of convergence insufficiency, measured by near point of convergence (NPC), or cognitive outcomes between those with symptomatic CI, and asymptomatic CI. However, NPC was greater for those with cognitive impairment (*x* = 18.4 cm), compared to those who were cognitively intact (*x* = 12.5 cm, *p* = 0.003).

**Conclusions:** Cognitive impairment commonly co-occurs in parkinsonian disorders with convergence insufficiency and is associated with significantly greater NPC distances. Clinicians should have a high index of suspicion for cognitive impairment in patients with objective findings of convergence insufficiency, whether symptomatic or not. Further investigation of convergence insufficiency in relationship to cognitive impairment in parkinsonian disorders is warranted, as there may be a shared mechanism of dysfunction.

## Introduction

Disjunctive, binocular eye movements performed to maintain single vision during near target viewing is referred to as *convergence*. Parkinsonian disorders frequently result in impaired convergence, which is a cause of disabling visual dysfunction, and decreased quality of life (1). Most commonly, convergence insufficiency (CI) presents with symptoms of double or blurred vision while reading or performing near tasks. Interestingly, one group found cognitive complaints to be more common in patients with Parkinson's disease (PD) who reported diplopia (2), but the relationship between cognitive impairment and symptomatic convergence insufficiency in parkinsonian disorders has not been previously investigated.

During binocular viewing, efferent and afferent visual pathways work together and rely on neurons within the brain stem, subcortical regions (i.e., the basal ganglia and thalamus), and posterior parietal and frontal cortical regions for an unambiguous, single, and steady view of the world. Convergence insufficiency has the potential to result from impaired subcortical and/or cortical function since, beyond the midbrain, it has been postulated that reflexive convergence that is stimulus bound is controlled by posterior parietal regions while volitional convergence is controlled by frontal cortical regions (3). We hypothesized that patients with parkinsonian conditions and CI were more likely to have cognitive impairment if they were symptomatic from CI.

## Materials and Methods

### Procedure

After IRB approval, records from the multidisciplinary neuro-ophthalmology/movement disorders clinic at the University of Colorado Anschutz Medical Campus from 2015 to 2017 were retrospectively reviewed for patients with CI in the setting of parkinsonian disorders. Fifty-five patients were identified on initial chart review, 31 of whom were excluded for the following reasons: near-point of convergence not measured (*n* = 18), exam inconsistent with CI (*n* = 8), ataxia rather than primarily parkinsonian presentation (*n* = 3), visual acuity worse than 20/40 in either eye (*n* = 1), cognitive impairment too severe to cooperate with ophthalmologic exam (*n* = 1).

Convergence insufficiency was defined by objective measures, including a near point of convergence (NPC) of ≥10 cm or a near/distance disparity of ≥10 prism diopters of an exotropia and is measured routinely in neuro-ophthalmology clinics for patients with parkinsonian disorders. In patients who required multiple trials for NPC testing, the smallest of the measured values was used. The presence of CI, the NPC in centimeters, and subjective reports of visual symptoms of CI (i.e., near tasks symptoms of blurred or double vision, eye strain, or reading complaints symptomatic of CI in the absence of alternative afferent, efferent, or cortical processing dysfunction on examination that could account for these symptoms) were recorded from the medical record. Demographics and clinical characteristics, including age, sex, parkinsonian diagnosis, disease duration, motor examination [using the Unified Parkinson's Disease Rating Scale (UPDRS)], presence of deep brain stimulation (DBS), and medication usage were also collected from the medical record. Patients independently completed the Self-Administered Gerocognitive Examination (SAGE) (4, 5) as a global cognitive screening measure after their office visits as part of their routine clinical care. The SAGE was scored and interpreted by a behavioral neurologist (S.K.H) who was blinded to the presence of visual complaints and neuro-ophthalmological examination results. Subjective cognitive complaints and years of education were captured from the demographic section of the SAGE.

### Statistical Analysis

The purpose of the current study was to explore potential connections between objective convergence insufficiency and cognitive impairment and generate pilot data for planning future studies; thus, no power calculations were performed. Means and standard deviations for the demographic and clinical characteristics of interest were calculated for the overall cohort, as well as for the symptomatic and asymptomatic CI groups. The cohort was also split into cognitively impaired and cognitive normal groups, based on SAGE score. Student's *t*-tests or proportion tests, as appropriate, were performed to compare these variables between groups. Logistic regression models were built, with cognitive impairment (defined as SAGE score <17) as the dependent binary outcome and symptomatic CI status or NPC as explanatory variables. Pre-selected covariates included age, years of education, and disease duration, which are independently associated with cognitive impairment. The hypotheses that symptomatic CI status and NPC would be associated with cognitive impairment were tested. Statistical analyses were performed using the Stata statistical software package (6).

## Results

Twenty-four patients were identified with parkinsonian disorders, convergence insufficiency, and visual complaints, who had undergone complete neuro-ophthalmologic evaluation and had SAGE data available. Eighteen had PD, five had progressive supranuclear palsy (PSP), and one had multiple system atrophy (MSA). 70.8% percent had visual symptoms attributed to convergence insufficiency (*N* = 17), and the other 29.2% (*N* = 7) had asymptomatic CI with visual complaints due to other causes (e.g., exotropia, saccadic deficiency, dry eye). Seventy-five percent reported subjective cognitive complaints and 54.1% met SAGE criteria for cognitive impairment (total score <17).

Comparison of symptomatic and asymptomatic patients with CI revealed no significant differences in demographic variables, disease duration, motor symptoms, presence of deep brain stimulation, medication usage, near point of convergence, or cognitive outcomes (Table 1). Comparison of cognitively impaired to cognitively normal patients demonstrated significant differences in subjective cognitive complaints (*p* = 0.03), use of cognitive enhancing medications (*p* = 0.04), and total SAGE scores (*p* = 0.006) as expected, but also revealed a significant difference in NPC between the groups (*p* = 0.003; Table 2). No other variables, including disease duration (mean difference 1.8 years, *p* = 0.63), were significantly different between cognitive groups.

**Table 1:** Demographic and clinical characteristics by convergence insufficiency (CI) status.

	**All participants** **(*n* = 24)**	**Symptomatic CI** **(*n* = 17)**	**Asymptomatic CI** **(*n* = 7)**	***p*-value**
Age *(years)*	71.3 (5.6)	71 (6.2)	72 (4.0)	0.70
Sex *(% female)*	41.7%	47.1%	28.6%	0.40
Education *(years)*	15.8 (2.4)	15.5 (2.3)	16.3 (2.5)	0.49
Neurological Diagnosis (*n*)				
PD	18	14	4	
PSP	5	3	2	
MSA	1	0	1	
Disease duration *(years)*	9.1 (8.6)	10.2 (9.5)	6.4 (5.7)	0.34
UPDRS Part III score	29 (11.7)	27.8 (11.4)	32.5 (12.6)	0.40
Presence of dyskinesia *(% with)*	16.7%	23.5%	0%	0.19
Medication use *(% taking)*				
Amantadine	16.7%	23.5%	0%	0.19
MAO-B Inhibitor	37.5%	41.2%	28.6%	0.69
Trihexiphenidyl	4.2%	5.9%	0%	0.54
Donepezil	16.7%	11.8%	28.6%	0.26
Presence of DBS *(% with)*	20.8%	23.5%	14.3%	0.70
NPC *(in cm)*	15.7 (5.1)	16.4 (5.5)	14.1 (3.9)	0.35
Subjective cognitive complaints *(% reporting)*	75%	76.5%	71.4%	0.80
Cognitive impairment *(% with)*	54.1%	47%	71.4%	0.23
Total SAGE score *(max 22)*	16.5 (5.0)	17.0 (1.1)	15.4 (6.0)	0.50

*Data presented as mean (SD) unless otherwise noted. CI, convergence insufficiency; PD, Parkinson's disease; PSP, Progressive supranuclear palsy; MSA, Multiple system atrophy; UPDRS, Unified Parkinson's Disease Rating Scale; DBS, Deep brain stimulation; NPC, near point of convergence; SAGE, Self-Administered Gerocognitive Exam*.

**Table 2:** Demographic and clinical characteristics by cognitive impairment status.

	**Cognitively impaired** **(*n* = 13)**	**Cognitively normal** **(*n* = 11)**	***p*-value**
Age *(years)*	69.8 (5.9)	73.1 (4.9)	0.15
Sex *(% female)*	38.5%	45.5%	0.73
Education *(years)*	15.0 (2.6)	16.6 (1.7)	0.09
Neurological diagnosis			
PD	10	8	
PSP	2	3	
MSA	1	0	
Disease duration *(years)*	8.3 (5.7)	10.1 (11.5)	0.63
UPDRS Part III score	28.2 (11.4)	29.9 (12.4)	0.73
Presence of dyskinesia *(% with)*	15.4%	18.1%	0.87
Medication use *(% taking)*			
Amantadine	15.4%	18.2%	0.87
MAO-B Inhibitor	30.8%	36.4%	0.48
Trihexiphenidyl	7.7%	0%	0.35
Donepezil	30.8%	0%	**0.04**
Presence of DBS *(% with)*	30.8%	9.1%	0.14
NPC *(in cm)*	18.4 (4.3)	12.5 (4.3)	**0.003**
Symptomatic CI	61.5%	81.8%	0.23
Subjective cognitive complaints *(% reporting)*	92.3%	54.5%	**0.03**
Total SAGE score *(max 22)*	14.1 (3.6)	19.5 (4.9)	**0.006**

*Data presented as mean (SD) unless otherwise noted. CI, convergence insufficiency; PD, Parkinson's disease; PSP, Progressive supranuclear palsy; MSA, Multiple system atrophy; UPDRS, Unified Parkinson's Disease Rating Scale; DBS, Deep brain stimulation; NPC, Near point of convergence; SAGE, Self-Administered Gerocognitive Exam. Bold values indicates p < 0.05*.

Logistic regression models demonstrated no significant relationship between symptomatic CI status and cognitive impairment (*p* = 0.29). However, there was a significant relationship between NPC and cognitive impairment, using linear regression, even when controlling for age, education, and disease duration (*p* = 0.038).

## Discussion

Given that cortical and subcortical regions are important for the maintenance of fusion of two retinal images, in addition to the brainstem, we hypothesized that patients with parkinsonism and symptomatic convergence insufficiency would be more likely to have cognitive impairment than those with asymptomatic CI, but our findings did not support this hypothesis. However, there was a high prevalence of cognitive impairment in all parkinsonian participants with CI. While the likelihood of dementia in PD increases with age and disease duration, estimates of dementia prevalence range widely, between 24 and 80% (7), and there is even less consistency regarding rates of dementia in atypical parkinsonian syndromes. Our cohort did have relatively long disease durations at the time of chart review (mean 9.1 years), and therefore were at higher risk of cognitive impairment. Yet, there was no significant difference in disease duration between those who were cognitively impaired and those who were not. We maintain that the severity of convergence insufficiency may serve as an additional marker for cognitive impairment in parkinsonian conditions, separate from age, and disease duration.

Previous investigations support visual dysfunction as an early marker of cognitive impairment in PD or PSP, including abnormalities on measures of color vision (8), intersecting pentagon copy (9), and visual exploration (10), as well as occipitoparietal hypometabolism on FDG-PET (11). In fact, Anang et al. (8) found that abnormal color vision increased dementia risk in PD to a greater degree than any motor or non-motor variable except for the presence of orthostatic systolic blood pressure drop of >10 mm Hg. Despite the fact that supranuclear palsy is a cardinal feature of PSP, up to 60% develop diplopia (12), and diplopia due to CI is very common in PSP. Tau deposition correlates to measures cognitive impairment (13), but available clinical data regarding visual dysfunction and dementia in PSP are lacking.

Limitations of this pilot study include the small sample size and the use of a self-administered cognitive screening measure as the primary cognitive outcome. Although the SAGE has been validated in Alzheimer's disease (5), it has not been specifically validated in parkinsonian disorders. Furthermore, the reliance on participants' subjective report of visual symptoms that could be attributed to CI for determination of symptomatic status is a relative limitation, though we attempted to restrict inclusion to those without potential alternative causes for their visual symptoms, based on thorough neuro-ophthalmological examination. The lack of a control group with parkinsonian disorders but without convergence insufficiency also limits the conclusions that can be drawn regarding the relationship between CI and cognition. In addition, the inclusion of typical (PD) and atypical (PSP, MSA) parkinsonian disorders in the same analysis could be problematic; our sample size was too small to perform sub-group comparisons. Conversely, we did not include all atypical parkinsonian disorders, namely dementia with Lewy bodies (DLB) and corticobasal syndrome (CBS), in this analysis, given lack of patients with these diagnoses who met our inclusion criteria. However, our goal was to determine whether the presence of symptomatic CI was more likely to be associated with cognitive impairment in any parkinsonian syndrome given recent findings associating cognitive complaints and diplopia in PD (2) and the overlap of signs and symptoms in parkinsonian disorders. Based on these initial findings, we are planning a larger study to include patients with parkinsonian disorders, typical and atypical, with and without CI to better examine the rate of co-occurrence of convergence insufficiency and cognitive impairment, as defined by more complete neuropsychological assessment, as well as their relative severities. Our goal is to better understand the temporal and mechanistic underpinnings of our initial observations in this population, in order to better diagnose cognitive impairment in parkinsonian conditions.

In conclusion, our findings support the need for further investigation into the onset of convergence insufficiency in relationship to cognitive impairment in parkinsonian disorders, and clinicians should be aware that the severity of CI in parkinsonian conditions is associated with cognitive impairment and patients should be screened or referred accordingly.

## Data Availability

The datasets generated for this study are available on request to the corresponding author.

## Ethics Statement

This study protocol was approved by the Colorado Multiple Institutional Review Board and granted exempt status (Category 4). Formal written informed consent was not required, as only de-identified information was collected, subjects were not contacted by the investigators, and there was no re-identification of subjects.

## Author Contributions

SH and VP contributed to the conception and design of the study. EV and SH performed chart review. SH performed the statistical analysis and wrote the first draft of the manuscript. VP and EV wrote sections of the manuscript. All authors contributed to manuscript revision, read, and approved the submitted version.

### Conflict of Interest Statement

SH has served on an advisory board for Acadia Pharmaceuticals. The remaining authors declare that the research was conducted in the absence of any commercial or financial relationships that could be construed as a potential conflict of interest.
